# Effect of Auditory Predictability on the Human Peripheral Auditory System

**DOI:** 10.3389/fnins.2020.00362

**Published:** 2020-04-15

**Authors:** Lars Riecke, Irina-Andreea Marianu, Federico De Martino

**Affiliations:** ^1^Department of Cognitive Neuroscience, Faculty of Psychology and Neuroscience, Maastricht University, Maastricht, Netherlands; ^2^Center for Magnetic Resonance Research, University of Minnesota, Minneapolis, MN, United States

**Keywords:** auditory attention, auditory efferent, prediction, expectancy, cochlea, electroencephalography, otoacoustic emission

## Abstract

Auditory perception is facilitated by prior knowledge about the statistics of the acoustic environment. Predictions about upcoming auditory stimuli are processed at various stages along the human auditory pathway, including the cortex and midbrain. Whether such auditory predictions are processed also at hierarchically lower stages—in the peripheral auditory system—is unclear. To address this question, we assessed outer hair cell (OHC) activity in response to isochronous tone sequences and varied the predictability and behavioral relevance of the individual tones (by manipulating tone-to-tone probabilities and the human participants’ task, respectively). We found that predictability alters the amplitude of distortion-product otoacoustic emissions (DPOAEs, a measure of OHC activity) in a manner that depends on the behavioral relevance of the tones. Simultaneously recorded cortical responses showed a significant effect of both predictability and behavioral relevance of the tones, indicating that their experimental manipulations were effective in central auditory processing stages. Our results provide evidence for a top-down effect on the processing of auditory predictability in the human peripheral auditory system, in line with previous studies showing peripheral effects of auditory attention.

## Introduction

Many socially relevant sounds in our natural environment arise from acoustic signals that have characteristic, regular spectral-temporal structures. The melody and rhythm of music, for instance, arise from specific spectral and temporal relations among the individual notes. Such a regular structure renders the constituent acoustic elements more predictable (in both time and spectral content), and human listeners can exploit this predictability to process and perceive the acoustic input more effectively. For example, prior knowledge of the pitch of an upcoming tone has been shown to facilitate perceptual detection of this tone in noise (Hafter et al., 1993). Prior pitch cues, if valid, also improve listeners’ judgments of the pitch, duration, and intensity of tones presented in melodic contexts (Dowling et al., 1987) or isolation (Mondor and Bregman, 1994; Ward and Mori, 1996). How the brain implements auditory predictions has been investigated extensively over the last decade. The common view (e.g., Clark, 2013) is that the brain aims to match ‘bottom-up’ acoustic input with ‘top-down’ auditory predictions at multiple levels of the auditory processing hierarchy by generating and dynamically updating the neural activity patterns that upcoming acoustic inputs are expected to evoke. This predictive coding theory (Rao and Ballard, 1999; Friston, 2005) has been supported by a large number of human studies showing evidence for predictive processing in the auditory cortex (reviews: Bendixen et al., 2012; Heilbron and Chait, 2018) and hierarchically lower, subcortical processing stages, including the medial geniculate body and the inferior colliculus (Slabu et al., 2012; Cacciaglia et al., 2015) (reviews: Winkler et al., 2009; Grimm and Escera, 2012; Chandrasekaran et al., 2014). It is still unclear whether predictive processing occurs also at the lowest stage of auditory processing, in the peripheral auditory system.

The peripheral auditory system receives top-down feedback from the central auditory system via the medial olivocochlear (MOC) efferent system (Huffman and Henson, 1990; Lopez-Poveda, 2018). The MOC system is a network of neurons located in the medial part of the superior olivary complex in the brainstem that receives ascending input from the cochlear nucleus and descending input via corticofugal projections. Efferent MOC fibers project to outer hair cells (OHCs) in the cochlea and activation of these fibers alters OHC activity, effectively reducing cochlear gain (reviews: Guinan, 2006, 2018). Efferent MOC-fiber activity can be modulated in a ‘top-down’ manner: electric microstimulation or deactivation of the auditory cortex alters OHC activity as measured with cochlear microphonics or otoacoustic emissions (OAEs) (Perrot et al., 2006; Dragicevic et al., 2015; Terreros and Delano, 2015; Jager and Kossl, 2016). Similarly, changes in arousal or endogenous (inter- or intramodal) attention may lead to OHC-activity changes as measured with OAEs (Puel et al., 1988; Froehlich et al., 1990, 1993; Giard et al., 1994; Ferber-Viart et al., 1995; Maison et al., 2001; de Boer and Thornton, 2007; Harkrider and Bowers, 2009; Smith et al., 2012; Srinivasan et al., 2012, 2014; Walsh et al., 2014, 2015; Wittekindt et al., 2014; Smith and Cone, 2015), although the existence and direction of these top-down attention effects are still debated (Picton et al., 1971; Avan and Bonfils, 1992; Michie et al., 1996; Beim et al., 2018, 2019; Francis et al., 2018; Lopez-Poveda, 2018).

Given that the efferent MOC system may reflect the state of endogenous attention, it might be able to reflect also the presence of auditory predictions generated in the central auditory system. Indeed, sectioning the efferent MOC fibers in humans impairs the aforementioned facilitating effect of pitch cues on tone-in-noise detection (Scharf et al., 1997). Animal electrophysiology findings further show that auditory-nerve fibers adapt to the statistics of acoustic input (Joris and Yin, 1992; Wen et al., 2009). These findings suggest that the peripheral auditory system may play a role in auditory predictions.

In the present study, we tested the hypothesis that auditory predictions are processed in the human auditory peripheral system. We presented 22 human listeners with isochronous complex-tone sequences and assessed tone-evoked OHC activity by measuring distortion-product OAEs (DPOAEs). To induce variations in auditory prediction we varied the predictability of the individual tones within a sequence (by manipulating tone-to-tone probabilities and keeping their acoustic properties constant) while concomitantly manipulating their behavioral relevance (by changing the listeners’ task). To check for the effect of our manipulation at central auditory processing stages, we simultaneously measured cortical tone-evoked activity (using electroencephalography, EEG) and behavioral auditory-detection performance. According to our hypothesis, auditory predictions should alter both cortical and peripheral tone-evoked activity. We predicted that increases in auditory predictability would lead to significant changes in both DPOAE and EEG that are strongest when the tones are behaviorally relevant.

## Materials and Methods

### Participants

Twenty-two healthy volunteers (ages: 19–28 years, 15 females) participated in the study. They had normal hearing (see section “Procedure”) and normal or corrected-to-normal vision. Participants gave their written informed consent before taking part and were compensated for their participation. The experimental procedure was approved by the local research ethics committee (Ethical Review Committee Psychology and Neuroscience, Maastricht University).

### Stimuli and Tasks

#### Auditory Stimuli

**FIGURE 1:**
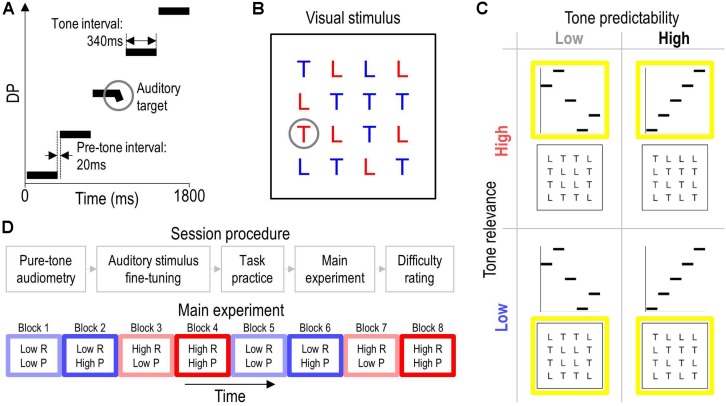
Stimuli, tasks, experimental design, and procedure. Panel **(A)** illustrates an exemplary auditory stimulus in a schematic spectrogram. The auditory stimuli comprised an isochronous sequence of five complex tones that were designed to evoke five consecutive OAEs at different DPs. The circle highlights an auditory target, which was a brief frequency glide at the end of one of tones. Panel **(B)** illustrates an exemplary visual stimulus. The circle highlights the visual target, which was the red letter ‘T’. Panel **(C)** illustrates the 2 × 2 experimental design. The predictability of the tones was varied by changing their frequency either pseudorandomly (low tone predictability) or monotonically (high tone predictability) across the auditory stimulus. The behavioral relevance of the tones was varied by having participants perform an auditory or visual target-detection task that required them to focus their attention on the tones (high tone relevance) or ignore them (low tone relevance), respectively. The yellow squares outline the task-relevant stimuli. Panel **(D)** sketches the overall procedure (top) and the task/stimulus protocol in the main experiment (bottom). Colored rectangles represent blocks of 50 trials from a given experimental condition (see **C**). Blue and red hue represents low and high tone-relevance condition (R), respectively. Light and saturated color represents low and high tone-predictability condition (P), respectively. Each condition was presented twice and the order of blocks was counterbalanced across participants. The direction of the tone sequence [ascending (see **A**) or descending] was fixed within high-predictability blocks and counterbalanced across the two presentations of these blocks.

Figure 1A illustrates an exemplary auditory stimulus. Auditory stimuli were isochronous sequences of five complex tones. Each tone lasted 340 ms and was preceded by a silent gap of 20 ms (pre-tone interval). Each tone was composed of two synchronous sinusoids—so-called primaries—with frequencies *f*1 and *f*2 = 1.22 × *f*1, resulting in a cubic distortion product, DP = 2 × *f*1 − *f*2. The intensity of the higher primary was defined as the test level. The intensity of the lower primary was always set 15 dB lower. These settings were chosen to facilitate elicitation of DPOAEs (Probst et al., 1991). Exact values for DP and test level were set individually for each participant (see section “Auditory Stimulus Fine-Tuning”). On half of the trials, a pseudorandomly chosen tone of the sequence contained a frequency glide during its final 40-ms portion, defining the auditory target. The glide was implemented by linearly changing the frequency of each primary by 20%.

#### Visual Stimuli

Figure 1B illustrates an exemplary visual stimulus. Visual stimuli were displays of the letters ‘L’ and ‘T’ on a PC screen. The letters were arranged pseudorandomly in a 4 × 4 matrix spanning a visual angle of approximately 5°. Each instance of the letter ‘L’ was colored in blue or red. Each instance of the letter ‘T’ was colored in blue, except for a single pseudorandomly chosen instance that was colored in red on half of the trials to define the visual target.

#### Tasks

On half of the trials, participants performed a behavioral task in the auditory sensory modality, for which they were instructed to attend to the auditory stimuli, ignore the visual stimuli, and detect the auditory target. On the other half, they performed the task in the visual modality, for which they received analogous instructions. Participants were informed that the probability of the target to occur was 50% for each task. They were further instructed to keep still and postpone any movement to a rest interval to avoid artifacts in the physiological recordings during stimulation intervals.

Trials contained a stimulation interval of 1.8 s followed by a response and rest interval of in total 7.1 s. The stimulation interval involved the synchronous presentation of an auditory and visual stimulus. Participants reported immediately after the stimulation whether they had perceived the designated target (‘yes’ response) or not (‘no’ response) by pressing a corresponding key with the index or middle finger of their right hand. After each response, they received visual feedback regarding response correctness and then relaxed until the next trial. Trials were preceded by silent gaps of 60 ms (pre-trial interval) during which the OAE recording started up (see section “OAE Recording”).

### Experimental Design

Figure 1C illustrates the experimental design. The study used a 2 × 2 within-subjects design. The independent variables were the predictability of the tones and the behavioral relevance of the tones. The manipulation of tone predictability was implemented by sequencing the five tones in an either highly predictable or unpredictable manner: Tone frequency was changed either monotonically (ascending or descending) or pseudorandomly across the tone sequence in each auditory stimulus, resulting in a local (tone-to-tone) probability of either 100% (*high-predictability condition*) or on average 45.7% (*low-predictability condition*; probability per position: 20, 25, 33, 50, and 100%). The increase in probability across positions within pseudorandom sequences resulted from permuting the five tones without repetition, which was done to distribute tone frequencies (and the associated DPOAE levels) equally across conditions.

The manipulation of the behavioral relevance of the tones was implemented by rendering the auditory stimuli either relevant or irrelevant for the participants’ behavioral goal. Participants were instructed to perform either the auditory task (*high tone-relevance condition*) or the visual task (*low tone-relevance condition*), which required them to focus their attention on the tones or ignore them, respectively (see section “Tasks”).

To further facilitate global (sequence-to-sequence) predictions in the high-predictability condition and avoid exhaustive task switching, trials belonging to a given condition were presented in blocks; see Figure 1D (bottom). The order of blocks was counterbalanced across participants to reduce potential carryover effects. Furthermore, the following variables were counterbalanced across trials within each block: the frequency and sequential position of the auditory target tone, the location of the visual target, and the sequential position of each tone (only for low-predictability blocks). The high- and low-relevance conditions were matched for stimulation.

### Procedure

Figure 1D (top) summarizes the session procedure, which was conducted in a sound-attenuated, electrically shielded chamber isolated from the experimenter. Participants were first screened for potential hearing loss, defined as a pure-tone hearing threshold above 25 dB HL at 0.75, 1, 1.5, 2, 3, or 4 kHz in the test ear.

#### Auditory Stimulus Fine-Tuning

Acoustically induced MOC effects tend to be more observable for higher signal-to-noise ratio (SNR) OAE measurements (Goodman et al., 2013) and for OAEs elicited at lower test levels (Ryan and Kemp, 1996; Veuillet et al., 1996), probably because OHCs apply less cochlear gain to lower-level acoustic input (Robles and Ruggero, 2001). To increase the likelihood of observing an auditory prediction effect, test levels in our study were chosen to be relatively low while being sufficiently high to reliably elicit DPOAEs of similar SNR across participants.

**FIGURE 2:**
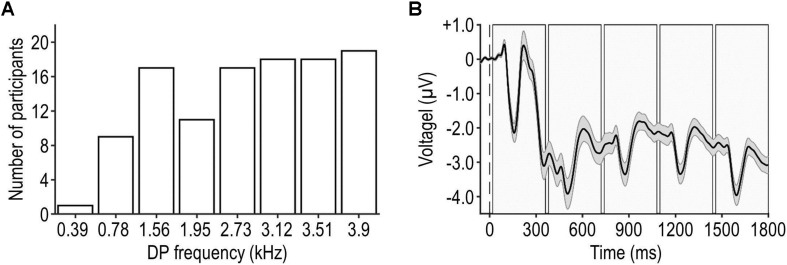
Frequency distribution of DP and grand-average EEG waveform. Panel **(A)** shows the frequency distribution of DPs tested in the main experiment. The most and least frequently tested DP was 3,900 and 390 Hz, respectively. Panel **(B)** illustrates participants’ average artifact-reduced EEG waveform during a trial (mean ± SEM across participants, represented by the black waveform and shaded surrounding area), pooled across frontal-central electrodes and all trials. The dashed vertical line represents the onset of the trial and the shaded rectangles represent the intervals of the individual tones.

To this end, a minimum test level and a set of most effective tones were defined individually for each participant as follows. First, DPOAEs were measured for eight different tones at a relatively high test level of 50 dB SPL. The frequencies of these tones were chosen to ensure that DPs (390, 780, 1,560, 1,950, 2,730, 3,120, 3,510, or 3,900 Hz) fell into distinct, resolvable frequency bins in the peripheral auditory system (Glasberg and Moore, 1990) and our OAE data analysis (see section “OAE Data Analysis”). Each DPOAE measurement involved 50 repetitions of a given tone presented at the same rate as the tones within the experimental stimuli. The tones that were found to evoke the five highest DPOAE SNRs (DPOAE level relative to the noise floor, see section “OAE Data Analysis”) were selected for the main experiment. The frequency distribution of the DPs that were selected for the main experiment is shown in Figure 2A.

Second, for the least effective selected tone (the one yielding the lowest DPOAE SNR), a DPOAE threshold was measured, defined as the minimum test level required for eliciting a DPOAE SNR of minimally 6 dB in half of the epochs. The threshold was measured with an adaptive staircase procedure and a one-down one-up tracking rule as follows: the test level was initially set to 35 dB SPL and then adaptively changed until eight reversals in the direction of change (from increasing to decreasing level or vice versa) occurred, after which the procedure terminated. The change size was gradually reduced from an initial 12 dB, to 6 dB (after the second reversal) and then to 3 dB (after the fourth reversal). The DPOAE threshold was computed as the average test level at the last four reversals.

Third, to ensure that all tones reliably elicited DPOAEs at a fixed low test level, DPOAEs were measured for the five selected tones as above, now using as test level the obtained DPOAE threshold. When a tone was observed to fail the criterion (DPOAE SNR ≥ 3 dB), the test level was increased by 3 dB and the measurement was repeated until the criterion was met. The test level resulting from this procedure was used for all tones in the main experiment and its average value was 30.7 ± 6.9 dB SPL (mean ± SD across participants, range: 18.5–48.5 dB SPL).

#### Experiment

Participants were familiarized with the stimuli and the auditory and visual targets, and they practiced the tasks before the experiment. Each experimental block contained 50 trials (corresponding to 250 tones), resulting in a block duration of 7.7 min. For each task condition (high or low tone-relevance), the low-predictability condition was presented in two blocks and each high-predictability condition (ascending or descending) was presented in a single block. This resulted in the presentation of eight blocks in total (Figure 1D bottom), corresponding to an overall number of 400 trials (corresponding to 2,000 tones) and an overall experiment duration of 61.3 min excluding breaks. Consecutive blocks were separated by short breaks terminated by the participant. Before and during each block, the current tone-frequency order (ascending, descending, or random) and the current task (auditory or visual) were visually indicated to the participant. After the experiment, participants rated the difficulty of each task on a five-point scale.

#### OAE Recording

The two primaries were presented to the participant’s right ear via two speakers mounted in a calibrated in-ear probe. OAEs were simultaneously recorded with a microphone in the probe. Auditory stimulation and recordings were sampled at 22.05 kHz and controlled using the Interacoustics Titan and Research Platform software (Interacoustics, Middelfart, Denmark) running on MATLAB (MathWorks, Natick, MA, United States). The Titan device requires the acquisition of several samples (lasting <60 ms) at the beginning of each recording for the recording level to settle. A tight probe fit (sealing of the ear canal) was ensured before each block using the Titan Suite software.

#### EEG Recording

Electroencephalography was recorded with 34 scalp electrodes positioned according to a modified 10–20 system (Easycap montage 11) and a reference electrode above the left mastoid, using BrainAmp amplifiers (Brain Products, Munich, Germany). Electrooculography was recorded with three additional electrodes positioned around the eyes. Inter-electrode impedances were kept below 20 kΩ. The EEG recordings were bandpass-filtered (cutoffs: 0.01 and 200 Hz, analog filter) and digitized with a sampling rate of 500 Hz.

### Data Analysis

The recorded data were analyzed using Matlab and SPSS software.

#### Behavioral Data Analysis

Behavioral data were analyzed as follows. Trials on which participants gave no response were discarded, which pertained to on average 7.9 ± 9.1 trials (mean ± SD across participants). Trials on which participants correctly reported the presence of the task-relevant target were labeled as hits. Trials on which they failed to report the absence of the task-relevant target were labeled as false alarms. Behavioral performance was assessed using the sensitivity index *d*′, which was obtained by subtracting the false-alarm rate from the hit rate, after correcting both measures for possible ceiling cases (Brown and White, 2005) and transforming the corrected measures to *z*-scores (Macmillan and Creelman, 1991).

#### OAE Data Analysis

Otoacoustic emissions data were analyzed as follows. First, the continuous data were segmented into 300-ms epochs, each corresponding to the tone interval excluding the final 40-ms portion. Second, epochs were classified as artifacts and discarded if the noise floor (defined below) exceeded its average value (mean across trials) by at least three standard deviations, which affected on average 16.8 ± 11.4 epochs (mean ± SD across participants). Third, epochs associated with the same tone frequency and belonging to the same condition were averaged. Fourth, sound-pressure level spectra were computed from the averaged epochs using the discrete Fourier transformation (number of points: 6615, resulting frequency resolution: 3.33 Hz) and the sensitivity curve of the OAE probe microphone. Fifth, a noise floor was extracted by averaging the level observed in a 20-Hz wide band centered on the frequency bin of the DP, excluding the latter bin. Sixth, the DPOAE level was extracted as the magnitude of the frequency bin of the DP. The DPOAE SNR was computed by subtracting the noise floor from the DPOAE level. Finally, DPOAE level and SNR were averaged across tone frequencies. For control analyses, physiological ear-canal noise was extracted as the broadband (390–3,900 Hz, corresponding to the tested DP range) sound level during the pre-tone interval, pooled across tone frequencies and positions.

#### EEG Data Analysis

Eelectroencephalography data were analyzed using the EEGLAB 14.1.2 toolbox (Delorme and Makeig, 2004) as follows. First, the eight blocks of recordings were concatenated. Second, the data were re-referenced to an average reference. Third, a bandpass filter was applied (cutoff frequencies: 0.5 and 30 Hz, FIR filter with zero phase shift, filter order: 3,300). Fourth, the continuous data were segmented into trials, each corresponding to the pre-trial interval and the stimulation interval. Fifth, a trial-specific baseline, defined as the average amplitude during the pre-trial interval, was subtracted from each trial. Sixth, temporally independent EEG components were extracted and artifactual components were identified and discarded based on visual inspection (e.g., Jaeger et al., 2018), which affected on average 34.9 ± 5.7% of the components (mean ± SD across participants). Figure 2B shows the artifact-reduced EEG waveform during the pre-trial and stimulation intervals, averaged across frontal-central EEG channels of interest (Fz, FC1, FCz, FC2, and Cz), trials, conditions, and participants. It can be seen that the neural response to the first tone was stronger than responses to subsequent tones within the same trial; this sensory adaptation was assumed to affect all conditions similarly. Seventh, trials were further segmented into epochs spanning the pre-tone interval and the tone interval. Note that long-latency components of the tone-evoked auditory-evoked potential (AEP) such as P3 could extend into consecutive epochs due to the short epoch duration and the relatively fast tone-presentation rate. While this methodological choice was suboptimal for the measurement of individual AEP components, it enabled efficient measurement of DPOAEs, which was the primary focus of our study. Eighth, epochs were classified as artifacts and discarded if the peak amplitude exceeded ±75 μV, which affected on average 11.1 ± 15.2 epochs (mean ± SD across participants). Ninth, an epoch-specific baseline, defined as the average amplitude during the pre-tone interval, was subtracted from each epoch. Tenth, epochs belonging to the same condition were averaged to obtain AEPs pooled across tone frequencies and positions, irrespective of the presence of the target (which was counterbalanced across tone frequencies and positions; see section “Experimental Design”). Eleventh, AEPs were averaged across the aforementioned EEG channels of interest. Finally, the peak amplitude of specific AEP components (P1, N1, and P2) originating in the cortex (Vaughan and Ritter, 1970) was defined within predefined time windows (50–90, 90–170, and 170–300 ms relative to the onset of the tone, respectively).

#### Correlation Analysis

To test whether the putative effects of auditory predictability on cortical and peripheral activity were functionally coupled, correlations between the predictability effects on OAE and EEG were assessed at the group level. To that end, the size of the predictability effect in the high tone-relevance condition, Δ, was extracted for each participant and measure (OAE and EEG) by subtracting the average value observed in the low-predictability condition from that observed in the high-predictability condition. Rank correlations between Δ_OAE_ and Δ_EEG_ were quantified using Kendall’s τ and statistically compared with zero.

#### Statistical Testing

Participants’ individual measures were submitted to second-level (random-effects) group analyses using parametric statistical tests (two-way ANOVAs and *t*-tests) for repeated measures. Assumptions of normality were verified with Kolmogorov–Smirnov tests, which did not reveal any significant deviation from normality. Violation of sphericity was compensated for using Greenhouse–Geisser correction. A significance criterion α = 0.05 was used and type-I error probabilities inflated by multiple comparisons were corrected by controlling the false-discovery rate (Benjamini and Hochberg, 1995). Reported summary statistics represent mean ± SEM across all participants unless stated otherwise.

## Results

### Behavioral Results

**FIGURE 3:**
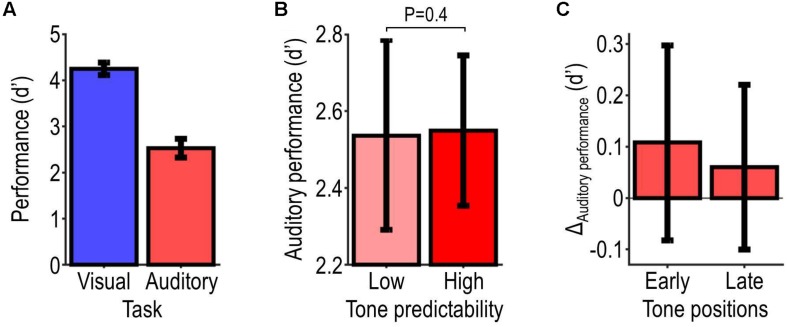
Behavioral results. Panel **(A)** illustrates participants’ overall performance as assessed with *d*′ for each target-detection task. Panel **(B)** illustrates performance on the auditory task for each tone-predictability condition, and the probability value associated with the effect of predictability. Panel **(C)** illustrates the predictability-related change in auditory performance (Δ_Auditory performance_ = high minus low-predictability condition) during the first and second half of the tone sequence. All data show summary statistics (mean ± SEM) across participants.

Figure 3 shows the behavioral results. Figure 3A shows participants’ overall performance as assessed with *d*′, which was on average 2.5 ± 0.2 in the auditory task and 4.3 ± 0.1 in the visual task (corresponding to response accuracies of 87.3 ± 2.4% and 98.2 ± 0.4%). Participants’ overall performance correlated significantly with their subjective ratings of the difficulty of the task (auditory task: τ = −0.57, *P* = 0.00095; visual task: τ = −0.44, *P* = 0.017). Figure 3B shows performance in the high-relevance (auditory task) condition, which was only slightly better for the high-predictability condition (average *d*′ = 2.55) than the low-predictability condition (average *d*′ = 2.54). This difference was not statistically significant (*t*_21_ = 0.074, *P* = 0.47). It was slightly larger for targets presented at early positions within the tone sequence vs. targets presented at later positions (first vs. second half of the tone sequence, balanced for tone frequencies; average difference: 0.048; Figure 3C). These null results indicate that the predictability of the tones did not improve auditory performance reliably across listeners.

### OAE Results

**FIGURE 4:**
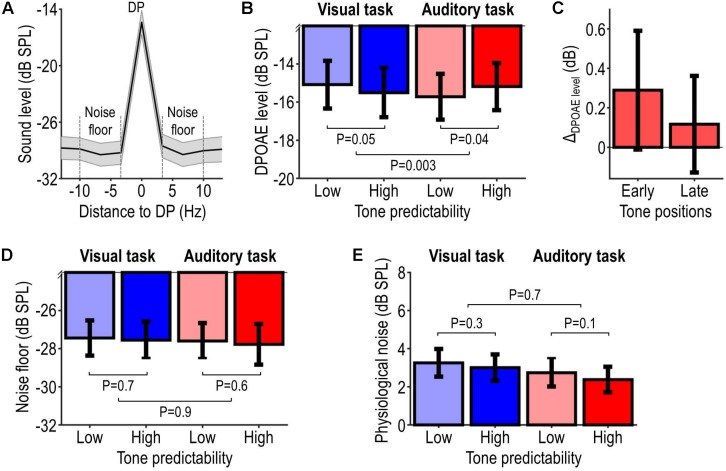
OAE results. Panel **(A)** illustrates the overall sound-pressure level spectrum of participants’ OAE recordings, averaged across tone frequencies and tone positions, and aligned with respect to the distortion product (DP) frequency. The DPOAE level was computed as the magnitude of the DP-frequency bin. The noise floor (delineated by the dashed lines) was computed as the average level in a 20-Hz wide band centered on, and excluding, the DP-frequency bin. The DPOAE SNR was computed by subtracting the noise floor from the DPOAE level and was observed to be on average 13.4 ± 4.8 dB (mean ± SD across participants). Panel **(B)** illustrates the DPOAE level pooled across tone frequencies and tone positions for each condition, and probability values associated with the effect of predictability (for each tone-relevance condition) and its modulation by tone relevance. Panel **(C)** illustrates the predictability-related change in DPOAE levels (Δ_DPOAE level_ = high minus low-predictability condition) elicited during the first and second half of the tone sequence in the auditory task. Panels **(D,E)** are analogous to panel **(B)**, but show, respectively, the level of the noise floor and the level of physiological ear-canal noise (instead of the DPOAE level). All data show summary statistics (mean ± SEM) across participants.

Figure 4 shows the OAE results. Figure 4A shows the grand average sound-pressure level spectrum of participants’ OAE recordings (pooled across tone frequencies and positions, and aligned to DP frequency). The DPOAE level and noise floor were on average −15.4 ± 6.0 dB SPL and −28.7 ± 5.4 dB SPL, which corresponds to an average DPOAE SNR of 13.4 ± 4.8 dB (mean ± SD across participants), a lower value than individual OAE SNR values (>20 dB) reported in previous attention studies using acoustic MOC-reflex elicitors (e.g., Beim et al., 2018). Figure 4B shows the DPOAE level for each condition. Task-relevant and task-irrelevant tones (auditory vs. visual task) elicited DPOAEs of overall similar level. When the tones were task-relevant, DPOAEs elicited by highly predictable tones were on average 0.54 ± 0.24 dB stronger than those elicited by less predictable tones. For task-irrelevant tones, a difference in the opposite direction (−0.42 ± 0.20 dB) was observed. Statistical analysis confirmed these observations, revealing no significant main effect of tone predictability (*F*_1_,_21_ = 0.12, *P* = 0.73) or tone relevance (*F*_1_,_21_ = 0.32, *P* = 0.58), and a significant interaction tone predictability × tone relevance (*F*_1_,_21_ = 11.50, *P* = 0.0028). *Post hoc* tests revealed a significant positive effect of tone predictability in the high relevance condition (*t*_21_ = 2.21, *P* = 0.038), and an opposite non-significant effect in the low relevance condition (*t*_21_ = −2.08, *P* = 0.051). Thus, these results provide evidence that the effect of auditory predictability on tone-evoked DPOAEs depends on the behavioral relevance of the tones.

The predictability-related difference in the high relevance condition, which we refer to as Δ_DPOAE level_, was slightly larger for tones presented at early positions within the tone sequence vs. tones presented at later positions within the same sequence (first vs. second half of the tone sequence, balanced for tone frequencies; average difference: 0.17 dB; Figure 4C).

To exclude that the OAE results could be explained by potential residual participant motion (Francis et al., 2018), the same statistical analyses as above were applied to the level of the noise floor and a measure of physiological ear-canal noise (the broadband sound level recorded during the pre-tone interval; see section “OAE Data Analysis”). Results are shown, respectively, in Figures 4D,E, revealing no significant interaction tone predictability × tone relevance (noise floor: *F*_1_,_21_ = 0.038, *P* = 0.85; physiological ear-canal noise: *F*_1_,_21_ = 0.13, *P* = 0.73) and no significant effect of tone predictability in either the high relevance condition (noise floor: *t*_21_ = −0.60, *P* = 0.55; physiological ear-canal noise: *t*_21_ = −1.73, *P* = 0.10) or low relevance condition (noise floor: *t*_21_ = −0.46, *P* = 0.65; physiological ear-canal noise: *t*_21_ = −1.17, *P* = 0.26). These statistical results indicate that the noise level did not differ significantly among the experimental conditions of interest. However, note that the non-significant results for the ear-canal noise may partially relate to the much shorter observation window (pre-tone interval < tone interval).

### EEG Results

**FIGURE 5:**
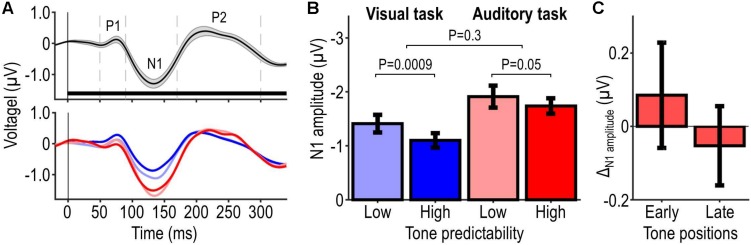
EEG results. Panel **(A)** illustrates participants’ average AEP evoked by single tones at frontal-central electrodes. The upper plot shows data pooled across tone frequencies, tone positions, and conditions, irrespective of the presence of the auditory target (mean ± SEM across participants, represented by the black waveform and shaded area). The horizontal bar represents the interval of a single tone and the dashed vertical lines delineate time windows from which peak amplitudes of P1, N1, and P2 were extracted. The lower plot shows the same data as the upper plot, but stratified for conditions (mean across participants). The different conditions are represented by different hue and brightness, which are labeled in **(B)**. Panel **(B)** illustrates N1 peak amplitude for each condition (mean ± SEM across participants), and probability values associated with the effect of predictability (for each tone-relevance condition) and its modulation by tone relevance. Panel **(C)** illustrates the predictability-related change in N1 amplitudes (Δ_N__1__amplitude_ = high minus low-predictability condition) elicited during the first and second half of the tone sequence in the auditory task.

Figure 5 shows the EEG results. Figure 5A shows participants’ AEP pooled across tone frequencies and positions, irrespective of the presence of the auditory target; the upper plot shows these data pooled across conditions and the lower plot shows them separately for each condition, revealing differences between conditions especially during the interval of the N1 component. Figure 5B shows the extracted N1 peak amplitude for each condition. Poorly predictable tones elicited overall stronger N1 amplitudes than highly predictable tones. Moreover, task-relevant tones elicited overall stronger N1 amplitudes than task-irrelevant tones. Statistical analysis of N1 peak amplitude confirmed these observations, revealing significant main effects of tone predictability (*F*_1_,_21_ = 12.19, *P* = 0.0022) and tone relevance (*F*_1_,_21_ = 19.16, *P* = 0.00026), and no significant interaction tone predictability × tone relevance (*F*_1_,_21_ = 1.11, *P* = 0.30). *Post hoc* tests showed a significant effect of tone predictability in the low tone-relevance condition (*t*_21_ = −3.58, *P* = 0.00087) and a corresponding non-significant effect in the high tone-relevance condition (*t*_21_ = −1.70, *P* = 0.052). The trend in the high tone-relevance condition, which we refer to as Δ_N__1__amplitude_, was slightly larger for tones presented at early positions within the tone sequence vs. tones presented at later positions within the same sequence (first vs. second half of the tone sequence, balanced for tone frequencies; average difference: 0.14 μV; Figure 5C). Analogous analyses of P1 and P2 revealed no significant result (all *P* > 0.05), except for a significant main effect of tone relevance on P1 (*F*_1_,_21_ = 5.86, *P* = 0.025). Thus, the EEG results indicate that the predictability and behavioral relevance of tones modulate the amplitudes of N1 and possibly longer-latency AEP components (see section “Discussion”).

### Combined OAE-EEG Results

**FIGURE 6:**
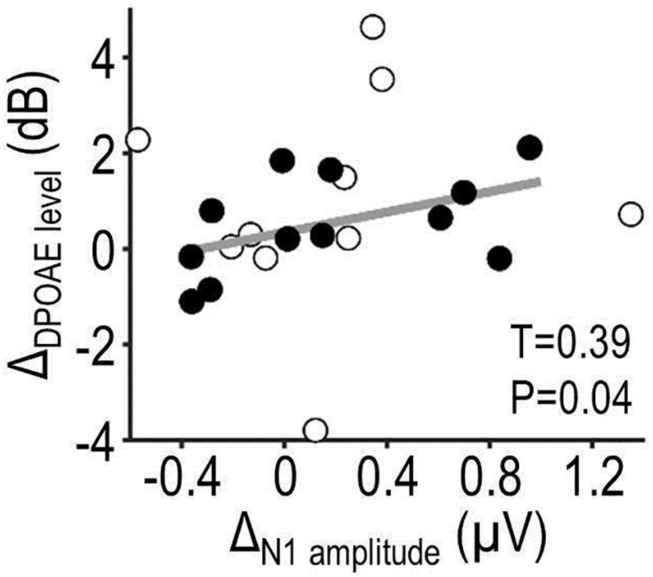
Combined OAE-EEG results. The scatterplot shows results from a correlation analysis testing for coupling between predictability effects on OAE and EEG (Δ_DPOAE level_ × Δ_N__1__amplitude_) during the auditory task. The analysis focused on data of participants whose auditory performance showed a qualitative benefit from predictability. Filled circles represent those participants; open circles represent remaining participants and two outliers. Correlation coefficient τ and P-value describe, respectively, the strength and statistical significance of the linear relationship (regression line, plotted in gray).

Figure 6 shows results from the correlation analysis testing for coupling between predictability effects on OAE and EEG (Δ_OAE_ and Δ_EEG_). The same measures as above were analyzed (DPOAE level and N1 peak amplitude). Initial analyses yielded no significant correlation between predictability effects on OAE, EEG, or behavior (all *P* > 0.05). Restricting the analysis to those participants who benefited from predictability as expected (better auditory performance for high vs. low-predictability condition; *N* = 14, of which two were outliers and rejected) revealed a significant positive correlation between Δ_DPOAE level_ and Δ_N__1__amplitude_ (τ = 0.39, *P* = 0.043). No significant correlation was observed in the low relevance condition (τ = 0.23, *P* = 0.14). These results suggest that predictability effects on DPOAEs and N1 amplitude might be functionally coupled.

## Discussion

The goal of our study was to test whether auditory predictions are processed in the human auditory peripheral system. To this end, we assessed whether OHCs are sensitive to the predictability of acoustic input by measuring DPOAE-level changes between a statistically structured sequence and a pseudorandom sequence composed of the same tones. Each tone had an equal probability of occurrence (20%), but the tone-to-tone (transitional) probabilities differed: only in the highly predictable sequence, all transitional probabilities were 100% and thus allowed listeners to generate strong auditory predictions. We observed that physically identical tones with different transitional probabilities can elicit different DPOAEs dependent on whether these tones are attended. Based on this result, we conclude that auditory predictability may influence OHC activity under auditory attention.

### Predictability-Induced Changes in Auditory Peripheral Responses

We observed a significantly larger predictability-induced DPOAE-level change in the high vs. low-relevance condition. A possible explanation for this effect is that the auditory, but not visual, task required participants to pay attention to the acoustic input and thereby encouraged them to actively generate and exploit predictions. The effect of our auditory task on predictability-induced DPOAE changes fits with previous OAE results showing effects of auditory attention on peripheral auditory processing, although these results are still debated (see section “Introduction”). Moreover, it is in line with studies showing a top-down attentional modulation of the effect of auditory predictability on cortical responses (reviews: Schroger et al., 2015; Heilbron and Chait, 2018). Probably only under auditory attention, predictive brain signals in our study (which we observed in cortex; see next section) were fed in a ‘top-down’ manner and gated to the peripheral auditory system. Alternatively, visual attention may have prevented these predictive signals from reaching the peripheral auditory system.

We further observed a positive effect of predictability on DPOAE levels in the high-relevance condition, which provides support for our hypothesis that auditory predictability influences peripheral auditory processing. Whether the observed effect reflects a potential cochlear amplification of actively anticipated input or an attenuation of this input cannot be disambiguated based on our DPOAE data. Efferent MOC signals may alter the amplitudes and phases of the two DPOAE components (primaries) by different amounts, which can result in either positive or negative DPOAE-level changes depending on the specific primary frequencies (Guinan, 2006)—an ambiguity that might explain why some previous DPOAE studies observed opposite effects of auditory attention (Smith et al., 2012; Srinivasan et al., 2012, 2014; Wittekindt et al., 2014). Future studies may disambiguate the direction of peripheral predictability effects by measuring, e.g., stimulus-frequency OAEs (SFOAEs), which require only a single test frequency but otherwise more sophisticated methods (Guinan, 2006; Lopez-Poveda, 2018). In sum, our DPOAE results suggest that OHCs may be sensitive to auditory predictability when the listener is paying top-down attention to the acoustic input.

### Predictability-Induced Changes in Cortical Responses

We observed that highly predictable tones evoke significantly smaller N1 amplitudes than poorly predictable tones, which is in line with previous EEG results showing a suppressive effect of auditory predictability on N1 (Schafer and Marcus, 1973; Martikainen et al., 2005; Baess et al., 2011). Given that listeners in our study could generate accurate predictions only in the high-predictability condition, the suppressive effect on N1 may reflect a cortical signal of fulfilled predictions (a ‘match’ signal), specifically the so-called repetition positivity (RP) (Bendixen et al., 2012). The auditory RP is an attenuation of auditory-evoked responses over the frontocentral scalp in a latency range from 50 to 250 ms. It increases with the number of stimulus repetitions (Bendixen et al., 2008) and the predictability of these stimuli (Costa-Faidella et al., 2011; Todorovic et al., 2011) even when attention is diverted away from them (Haenschel et al., 2005). The early portion (40–60 ms) of the RP is mostly affected by stimulus repetitions, whereas its later portion (100–200 ms) is more sensitive to predictability (Todorovic and de Lange, 2012). Thus, the suppressive effect on N1 in our study may reflect auditory predictions rather than sensory adaptation (Heilbron and Chait, 2018). However, the observed suppression may also reflect frequency-specific adaptation, which was probably stronger in the high vs. low-predictability condition (due to overall smaller tone-to-tone frequency changes). While consecutive DPOAEs could not interact within single auditory filters in the peripheral auditory system (see section “Auditory Stimulus Fine-Tuning”), they might have done so within broader filters in the auditory cortex (Bartlett and Wang, 2005). It should be noted that the observed cortical suppression may have affected also much later AEPs. Owing to the fast tone-presentation rate, these later AEPs and consecutive N1 responses overlapped, making it difficult to disentangle them (see section “EEG Data Analysis”).

The observed suppressive effect presumably extends to the auditory brainstem, as suggested by corresponding predictability effects observed in an EEG study that measured auditory brainstem responses to auditory stimuli similar to ours (patterned vs. pseudorandom complex tone sequences) (Skoe et al., 2013). This would imply that our high-predictability condition induced an internal predictive model in the cortex (and possibly subcortex) that may have sent predictive signals ‘top-down’ to hierarchically lower auditory processing stages, especially when the listener paid attention to the acoustic input.

We did not observe cortical signals of violated predictions (‘mismatch’ signals), such as the mismatch negativity (Naatanen et al., 2001), probably because the high-predictability condition contained no regularity violation and the low-predictability condition did not allow generating highly accurate predictions. We further observed that behaviorally relevant tones evoked significantly larger N1 amplitudes than irrelevant tones, which is in line with previous EEG results showing an enhancing effect of auditory attention on N1 (e.g., Picton and Hillyard, 1974; Naatanen, 1982).

In sum, while our EEG results cannot disentangle effects on N1 and much later AEP components, they confirm that our experimental manipulations of predictability and behavioral relevance were effective in the cortex.

### Coupling Between Predictability Effects on Cortical and Peripheral Responses?

Our results suggest that predictability effects on DPOAE and N1 might be positively correlated. More specifically, listeners who showed larger predictability effects on DPOAE tended to show also larger predictability effects on N1 amplitude. Moreover, predictability-induced changes in DPOAE, N1, and auditory performance were qualitatively (non-significantly) stronger for tones presented early vs. late during a given tone sequence, which might reflect reduced effectiveness of our predictability manipulation toward the end of auditory stimuli (due to the blocked design and the fact that tone-to-tone probabilities in the low-predictability condition approximated those in the high-predictability condition at later tone positions; see section “Experimental Design”). Together, these observations might suggest that prediction effects on different auditory processing stages are functionally coupled, which would imply that the effects propagate through the auditory system. However, it should be noted that the interpretation of our correlation results needs to be treated with caution because these results are based on only a subset of participants (those who appeared to benefit from predictions).

Previous studies indeed found similar prediction effects in multiple stages of the central auditory processing hierarchy (see section “Introduction”). Whether these stages locally generate auditory predictions or inherit them ‘top-down’ from higher stages is still unclear (Escera and Malmierca, 2014; Malmierca et al., 2019), although auditory prediction effects seem to become stronger toward higher hierarchical stages (Gill et al., 2008; Rummell et al., 2016). It is further possible that functional coupling of cortical and peripheral top-down modulations is mediated by slow (<10 Hz) periodic brain signals, as suggested by recent DPOAE/EEG-oscillation findings on endogenous intermodal (audiovisual) attention (Dragicevic et al., 2019).

### No Significant Effect of Predictability on Behavioral Responses

We observed better auditory performance in the high vs. low-predictability condition, but this difference was only small and not statistically significant. Thus, apparently not all our participants made effective use of predictions, despite the fact that our auditory stimuli and task were designed to encourage the use of such predictions. A possible explanation for our null result is that the pitch-specific predictions that could be inferred from our highly predictable stimuli did not provide the most potent cue for detecting the auditory target (which was a sudden pitch change within a tone). Moreover, perceptual benefits from predictions may be more observable under conditions of sensory uncertainty or ambiguity (de Lange et al., 2018). Our auditory targets were not necessarily ambiguous or near participants’ perceptual detection threshold, implying that predictions derived from our stimuli were not optimally effective for these particular stimuli and our auditory task. In sum, our behavioral null result shows that our participants’ auditory perception did not reliably benefit from predictions. Future studies may achieve stronger predictability effects by using more ambiguous auditory stimuli and improved tasks that allow for stricter control of listeners’ use of predictions.

### Critical Considerations

The level of our auditory stimuli (on average ∼30 dB SPL) was probably sufficiently low to prevent elicitation of middle-ear muscle reflexes, but whether it was sufficiently high to elicit ipsilateral MOC reflexes, which seem to require levels of at least ∼30 dB SPL (Guinan et al., 2003), remains unclear. Even if not all tones in our study elicited MOC reflexes, it remains possible that corticofugal top-down signals modulated MOC efferent activity in the absence of an acoustic elicitor, as suggested by studies showing attention effects on OAEs without an acoustic MOC-reflex elicitor (Puel et al., 1988; Giard et al., 1994) (but see Picton et al., 1971). Future studies should measure OAEs at higher SNR and use contralateral acoustic noise to control MOC reflexes more effectively to increase the strength of top-down modulation of the MOC reflex.

The auditory targets in our auditory task (which were defined by the pitch of the two primaries) differed from the investigated DPOAE frequencies, similar to some previous OAE attention studies (Srinivasan et al., 2012, 2014; Wittekindt et al., 2014). It is unlikely that this difference largely reduced effect sizes in our study because acoustically evoked (Norman and Thornton, 1993; Lisowska et al., 2002) (review: Guinan, 2018) and attention-modulated (Srinivasan et al., 2012, 2014; Wittekindt et al., 2014; Beim et al., 2018) MOC reflexes show only little frequency tuning in humans.

It should be noted that our interpretation is constrained by our auditory stimulus design to predictions of pitch or spectral structure. Moreover, the high-predictability blocks involved exposure to fixed patterns over relatively long durations, which likely facilitated long-term learning of acoustic structure. Whether our results generalize to predictions of other features, such as timing, or to short-term (echoic) memory of acoustic structure remains to be investigated.

## Conclusion

Our study shows that intramodal top-down attention may modulate the processing of predictable spectral input in the peripheral auditory system, thereby adding support to current debate on the sensitivity of the human auditory peripheral system to top-down modulation (e.g., Beim et al., 2018; Lopez-Poveda, 2018). Our observations further indicate that predictability may affect auditory processing in the peripheral auditory system. Future studies may observe stronger predictability effects on peripheral auditory processing by measuring higher-SNR SFOAEs in the presence of an acoustic contralateral MOC-reflex elicitor and using more optimal (ambiguous) auditory stimuli.

## Data Availability Statement

The datasets generated for this study are available on request to the corresponding author.

## Ethics Statement

The studies involving human participants were reviewed and approved by Ethical Review Committee Psychology and Neuroscience, Maastricht University. The patients/participants provided their written informed consent to participate in this study.

## Author Contributions

LR, FD, and I-AM conceived the study. I-AM acquired the data. LR and I-AM performed the statistical analysis. LR wrote the first draft of the manuscript. All authors contributed to manuscript revision, read and approved the submitted version.

## Conflict of Interest

The authors declare that the research was conducted in the absence of any commercial or financial relationships that could be construed as a potential conflict of interest.
